# The Role of Hypothalamic Microglia in the Onset of Insulin Resistance and Type 2 Diabetes: A Neuro-Immune Perspective

**DOI:** 10.3390/ijms252313169

**Published:** 2024-12-07

**Authors:** Radwan Darwish, Yasmine Alcibahy, Shahd Bucheeri, Ashraf Albishtawi, Maya Tama, Jeevan Shetty, Alexandra E. Butler

**Affiliations:** 1 School of Medicine, Royal College of Surgeons in Ireland-Medical University of Bahrain (RCSI-MUB), Busaiteen 228, Bahrain; 22200493@rcsi-mub.com (R.D.); 21201313@rcsi-mub.com (Y.A.); 21200330@rcsi-mub.com (S.B.); 22200312@rcsi-mub.com (A.A.); 22200235@rcsi-mub.com (M.T.); 2 Department of Biochemistry, Royal College of Surgeons in Ireland-Medical University of Bahrain (RCSI-MUB), Busaiteen 228, Bahrain; jshetty@rcsi-mub.com; 3 School of Postgraduate Studies and Research, Royal College of Surgeons in Ireland-Medical University of Bahrain (RCSI-MUB), Busaiteen 228, Bahrain

**Keywords:** hypothalamus, neuroinflammation, microglia, microglial activation, type 2 diabetes mellitus, insulin resistance

## Abstract

Historically, microglial activation has been associated with diseases of a neurodegenerative and neuroinflammatory nature. Some, like Alzheimer’s disease, Parkinson’s disease, and multiple system atrophy, have been explored extensively, while others pertaining to metabolism not so much. However, emerging evidence points to hypothalamic inflammation mediated by microglia as a driver of metabolic dysregulations, particularly insulin resistance and type 2 diabetes mellitus. Here, we explore this connection further and examine pathways that underlie this relationship, including the IKKβ/NF-κβ, IRS-1/PI3K/Akt, mTOR-S6 Kinase, JAK/STAT, and PPAR-γ signaling pathways. We also investigate the role of non-coding RNAs, namely microRNAs and long non-coding RNAs, in insulin resistance related to neuroinflammation and their diagnostic and therapeutic potential. Finally, we explore therapeutics further, searching for both pharmacological and non-pharmacological interventions that can help mitigate microglial activation.

## 1. Background

Type 2 diabetes mellitus (T2DM) is a chronic metabolic disorder characterized by a combination of insulin resistance (IR) and a decline in insulin production due to loss of pancreatic β-cells [1,2]. According to the International Diabetes Federation (IDF), the global diabetes prevalence in individuals between the ages of 20 to 79 years was estimated to be 10.5% (536.6 million) in 2021 and has been projected to rise to 12.2% (783.2 million) in 2045 [3].

IR, a hallmark of T2DM, not only dysregulates glucose metabolism but also interferes with various brain functions, suggesting a complex interplay between metabolic disorders and neuroinflammation [4]. Recent evidence suggests an intimate relationship between obesity and hypothalamic microglial activation, initiated during the early stages of high-fat diets (HFDs) in rodents [5,6,7,8,9]. However, in the setting of T2DM, there is a paucity of data on hypothalamic neurocircuits.

Here, we seek to explore the explicit relationship between hypothalamic inflammation and microglial activation and systemic IR and T2DM, looking at the pathways that connect these two concepts and how they can be exploited in diagnostics and therapeutics.

## 2. Hypothalamic Function and Microglia

The central nervous system (CNS), particularly the hypothalamus, plays a significant role in metabolic regulation and homeostasis. Metabolic regulation is specifically controlled by the arcuate nucleus (ARC), which comprises two neuronal populations with antagonistic functions that operate on a presumably neurocircuitry basis, namely agouti-related peptide/neuropeptide-Y (AgRP/NPY) and proopiomelanocortin (POMC) [10].

AgRP/NPY is an orexigenic peptide responsible for hunger; AgRP antagonizes the actions of melanocortin-4 receptors (MC4Rs) and melanocortin-3 receptors (MC3Rs), therefore stimulating food intake [11,12]. Conversely, POMC neurons are responsible for inhibiting food intake and stimulating energy expenditure by releasing α-melanocyte-stimulating hormone (α-MSH), a potent agonist of MC4Rs and MC3Rs [11,13,14]. The production of α-MSH is stimulated by the coordinated release of leptin [12,15,16]. The reciprocal activities of AgRP/NPY and POMC regulate energy balance and coordinate metabolic control [10].

Microglia are the resident immune cells of the CNS [17]. First described by Nicolas Achucarro and Pio del Rio-Hortega in the early 20th century, they were thought to be inactive, only contributing to the brain neuroinflammatory response in a detrimental manner [18,19,20]. It was not until investigators imaged microglia that they discovered their varied roles in bodily functions, including, but not exclusive to, metabolic ones [21,22].

Since then, microglia have been recognized for their role in promoting neuronal injury or death in response to abnormal molecular patterns associated with neuronal degenerative disorders [23]. Histologically, the cardinal hallmark of hypothalamic inflammation is gliosis, characterized by microglial infiltration and astrocyte proliferation, eventually leading to glial scarring [24].

Microglia express common macrophage markers such as the CX3C motif chemokine receptor 1 (CX3CR1), colony-stimulating factor 1 receptor (CSF1R), cluster of differentiation molecule 11B (CD11b), F4/80, cluster of differentiation molecule 68 (CD68), ionized calcium-binding adaptor molecule 1 (Iba1), and cluster of differentiation molecule 45 (CD45), which are involved in inflammatory processes [25]. The severity of microglial responses directly correlates with the extent of neural injury, contributing to IR, synaptic dysfunction, and other neurophysiological changes. Hypothalamic microglia are quiescent under normal conditions but become particularly responsive to cytokines like interleukin-1β (IL-1β) and interleukin-6 (IL-6), which enhance pro-inflammatory activity [26,27].

## 3. Insulin Resistance and Type 2 Diabetes Mellitus

IR was first observed by Himsworth, who noted two distinct outcomes when injecting insulin into T2DM patients. Some responded with stable or decreased blood glucose, described as insulin-sensitive, while others presented with significantly increased blood glucose, described as insulin-insensitive [28].

IR is the reduced ability of insulin, either endogenous or exogenous, to affect an increase in glucose uptake and utilization [29]. IR is mainly present in the muscle and the liver [30,31,32], though it is also present in other organs, such as adipose tissue [33,34], the gastrointestinal tract [35], and kidneys [36].

In muscles, multiple factors contribute to IR, including defects in insulin signaling, glycogen synthesis, glucose phosphorylation, glucose transport, pyruvate dehydrogenase complex activity, and mitochondrial oxidative activity [30,37,38]. In the liver, the regulatory role of insulin in suppressing gluconeogenesis and promoting glycogenesis is impaired, leading to gluconeogenic activity [39,40,41] and contributing to fasting hyperglycemia [41,42,43].

Generally, IR shares an intimate bi-directional relationship with inflammation [44,45], as has been well described in adipose tissue [46]. Here, we explore what is known about the role that hypothalamic inflammation, mediated by microglia, plays in IR and T2DM.

## 4. Neuro-Immune Crosstalk in the Hypothalamus

**Figure 1:**
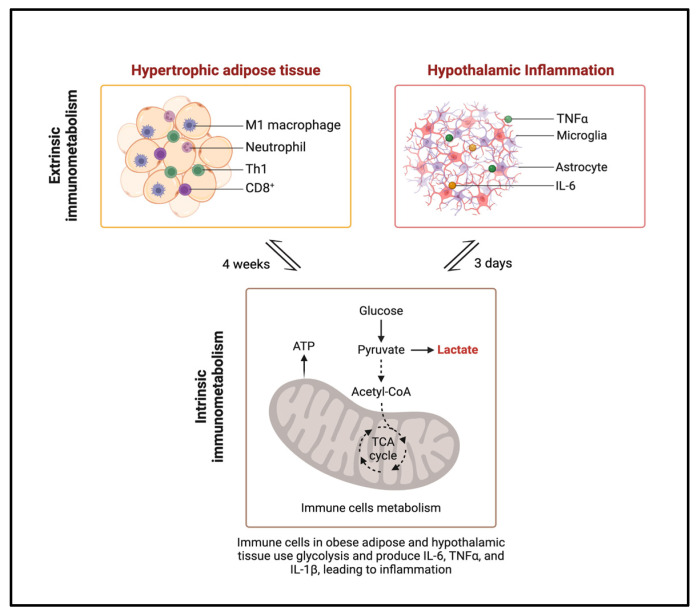
Extrinsic and intrinsic immunometabolic processes associated with adipose and hypothalamic inflammation.

In the hypothalamus, IR in AgRP/NPY and POMC neurons disrupts whole-body energy balance, leading to obesity through changes in feeding behavior, altered glucose metabolism, and increased fat breakdown [47]. These findings suggest that central IR can trigger systemic energy imbalances, which, through a cascade of downstream events, exacerbates IR in the brain (Figure 1).

Although the broader effects of impaired insulin signaling on microglial activation are not fully understood, available evidence suggests that microglia and astrocytes can undergo active gliosis, thereby promoting inflammatory responses in the presence of long-chain saturated fatty acids (SFAs) [48].

Similarly to macrophages in the peripheral immune system, microglia are activated by stress or injury to the CNS, releasing pro-inflammatory cytokines that affect peripheral organs [49,50].

A 2022 study, which investigated the effects of hyperinsulinemia on microglial function and neuroinflammation in 4-week-old C57BL/6J background male mice fed an HFD over 12 weeks, observed a positive correlation between the two. Hyperinsulinemia induced M1 microglial polarization, increasing neuroinflammation within the hypothalamus and beyond [51].

Previous evidence derived from rodent models demonstrated that as little as 1 to 3 days of HFD feeding can prompt inflammation in the hypothalamus, preceding any noticeable increase in body weight. This contrasts with the timeline of inflammation in adipose tissue, which typically arises after several weeks of HFD consumption and is generally associated with the onset of obesity [48].

The underlying mechanism of this neuroinflammation is unclear, but one purported theory suggests that insulin activates microglia, prompting the release of pro-inflammatory cytokines, including cyclooxygenase-2 (COX-2), interleukin-17 (IL-17), IL-1β, and IL-6 [27,52,53].

To investigate the potential impact of insulin on neuroinflammation, Spielman et al. utilized in vitro human cell culture models [54]. Findings revealed that both human astrocytes and microglia express the two isoforms of the insulin receptor (InsR), as well as the insulin-like growth factor (IGF)-1 receptor. These cells express InsR substrate (IRS)-1 and IRS-2, which are essential for insulin/IGF-1 signal transduction. At low nanomolar concentrations (10 pM–1 nM), insulin seemed to promote inflammation by increasing IL-8 secretion in activated microglia. However, this pro-inflammatory effect diminished at higher insulin levels (1 nM–1 μM).

Across a broader insulin concentration range (10 pM–1 μM), insulin mitigated the toxicity of activated human microglia and THP-1 monocytic cells toward neuronal cells. These results suggest that insulin modulates the inflammatory state of glial cells, selectively regulating their functions. This, in turn, could impact neuronal survival and help explain the association between T2DM and neuroinflammation.

Together with the previously described hypothalamic inflammatory state disrupting InsR signaling around the body [55], this creates a cyclic relationship, where systemic IR causes hypothalamic inflammation, which further exacerbates systemic IR. Given the detrimental nature of this relationship, we will explore specific pathways, both individually and in association with each other, to investigate the connection between hypothalamic inflammation and IR.

## 5. Mechanisms and Pathways

Several pathways have been implicated in the bi-directional relationship between IR and hypothalamic inflammation. These are the nuclear factor kappa-light-chain enhancer of activated B cells/inhibitory kappa B kinase beta (IKKβ/NF-κβ), insulin receptor substrate 1/phosphoinositide 3-kinase/protein kinase B (IRS-1/PI3K/Akt), mammalian target of rapamycin ribosomal protein S6 kinase (mTOR-S6 Kinase), Janus kinase/signal transducer and activator of transcription (JAK/STAT), and peroxisome proliferator-activated receptor gamma (PPAR-γ) signaling pathways.

### 5.1. IKKβ/NF-κβ Signaling Pathway

At the molecular level, the IKKβ/NF-κB inflammatory pathway plays a pivotal role in the onset and progression of hypothalamic IR. Studies have shown that suppressing IKKβ/NF-κB signaling in neurons, astrocytes, or microglia within the mediobasal hypothalamus protects against HFD-induced obesity, glucose intolerance, and IR in the hypothalamus [56,57,58].

**Figure 2:**
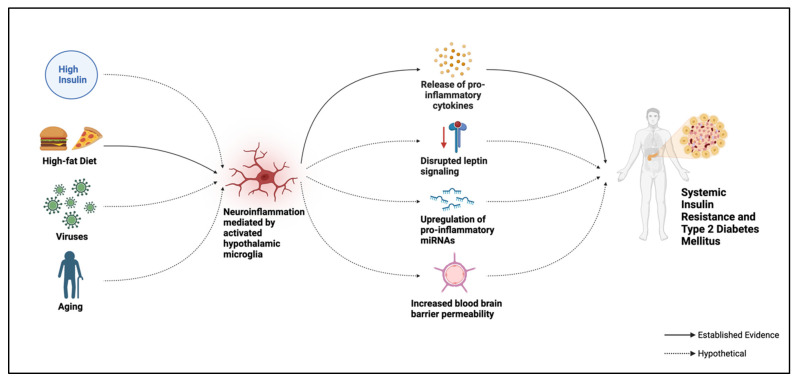
A hypothetical molecular scheme showing the relationship between neuroinflammation mediated by hypothalamic microglia and systemic insulin resistance and type 2 diabetes mellitus.

The elevated activity of NF-κB signaling in the hypothalamus of rodents on an HFD contributes to endoplasmic reticulum (ER) stress, exacerbating hypothalamic IR and accelerating the onset of both obesity and T2DM [56,59,60], as shown in Figure 2 below. NF-κB activation also drives the expression of the suppressor of cytokine signaling 3 (SOCS3) and 1 (SOCS1) in the hypothalamus. SOCS3 normally inhibits insulin signaling through two distinct mechanisms [61,62,63]. First, it impairs insulin-dependent phosphorylation of InsRs. Second, it promotes the degradation of InsR substrates 1 and 2 (IRS1/2) via the proteasome [56,64]. Similarly, SOCS1 interacts with other inflammatory modulators to fine-tune inflammatory responses [65]. Studies have shown that microglial and astrocyte cultures respond to inflammatory stimulants by expressing cytokine-induced Src homology 2 (SH2), protein (CIS), SOCS1, and SOCS3 as early as 30 min after stimulation [65,66,67]. It has also been reported that SOCS1 suppresses the JAK/STAT signaling pathway, thereby contributing to the resolution of inflammation in the hypothalamus [68,69,70]. Specifically, it suppresses signaling pathways activated by interferons and cytokines such as interleukin-2 (IL-2) and interleukin-4 (IL-4) and inhibits toll-like receptor 4 (TLR4) signaling, which is crucial for activating NF-κB [71,72,73,74]. SOCS1 also protects against the chronic low-grade inflammation linked to IR and T2DM [63,75,76]. Notably, it has been observed that SOCS1-deficient mice develop symptoms of severe systemic autoimmune and inflammatory disease, highlighting the critical role of SOCS1 in maintaining inflammatory balance [70,77,78,79].

Protein tyrosine phosphatase 1B (PTP-1B) is another key regulator that negatively impacts insulin signaling. HFD feeding upregulates PTP-1B in the hypothalamus, impairing insulin’s anorexigenic effects by dephosphorylating InsRs [80,81]. Mice with neuron-specific deletion of *PTP-1B* exhibit enhanced insulin sensitivity in the hypothalamus and are protected from HFD-induced obesity and related metabolic dysfunctions [82]. Ablation of the *PTP-1B* gene also reduces HFD-induced hypothalamic inflammation [80,83]. Taken together, these findings suggest that the IKKβ/NF-κB pathway, along with SOCS3, SOCS1, and PTP-1B, serve as a critical bridge between hypothalamic inflammation and IR.

### 5.2. IRS-1/PI3K/Akt Signaling Pathway

Recent studies show that microglia also have InsRs and respond to insulin in vitro [54]. When exposed to pathogen-associated molecules (PAMPs) like lipopolysaccharide (LPS), insulin activates the IRS-1/PI3K/Akt signaling pathway, enhancing microglial phagocytic activity [84].

In vivo, insulin consistently activates microglia and elevates COX-2/IL-1β expression in the hippocampus of young male rats but not aged rats [52]. This suggests that insulin can modify microglial behavior, with an excess potentiating a pro-inflammatory microglial state in young male rates. However, the microglial response to insulin is lost or perturbed during the aging process.

In obesity, increased blood–brain barrier (BBB) permeability may allow more insulin to enter the brain, contributing to neuronal IR, possibly through receptor desensitization, a process thought to worsen with aging [85,86]. Peripheral immune cells exposed to chronic insulin become insulin-resistant and display altered inflammatory responses, raising the question of whether microglia also develop IR and how this impacts their normal function.

### 5.3. mTOR-S6 Kinase Signaling Pathway

Chronic hyperinsulinemia and excessive nutrient intake activate the mammalian target of rapamycin (mTOR) and its downstream effector, p70 S6 kinase (S6K) [87]. This activation causes the serine phosphorylation of IRS-1, creating a negative feedback loop that inhibits insulin signaling [88].

In the hypothalamus, the mTOR/S6K pathway is highly responsive to HFDs. S6K becomes activated as early as one day within initiation of HFD consumption, leading to decreased IRS-1 tyrosine phosphorylation and reduced Akt activation, both of which contribute to impaired insulin signaling [89].

The overactivation of hypothalamic S6K, induced via viral vectors, triggers IR both locally in the hypothalamus and systemically. In contrast, inhibiting the mTOR/S6K pathway in the hypothalamus effectively reverses HFD-induced IR, and these improvements in glucose metabolism occur independent of changes in body weight [87].

Interestingly, the effects of S6K on food intake vary from those on glucose metabolism. While mTOR/S6K negatively regulates insulin signaling, it simultaneously suppresses food intake in the hypothalamus. This suggests that the mTOR/S6K pathway serves as a nutritional sensor independent of its role as an insulin regulator [90].

### 5.4. JAK/STAT Signaling Pathway

Leptin, produced by adipocytes, influences the hypothalamus to control hunger and energy expenditure. When leptin or its receptor is absent, as seen in the ob/ob and db/db mouse models, it leads to extreme weight gain and IR [91].

Leptin exerts its effects by binding to the leptin receptor (LepRb/ObRb), activating JAK2 through auto-phosphorylation. This then leads to the phosphorylation of specific tyrosine residues (Y985, Y1077, Y1138) on the receptor, allowing for the recruitment and activation of signal transducers and activators of the transcription (STAT) pathway, namely STAT3 and STAT4 [92,93,94].

**Figure 3:**
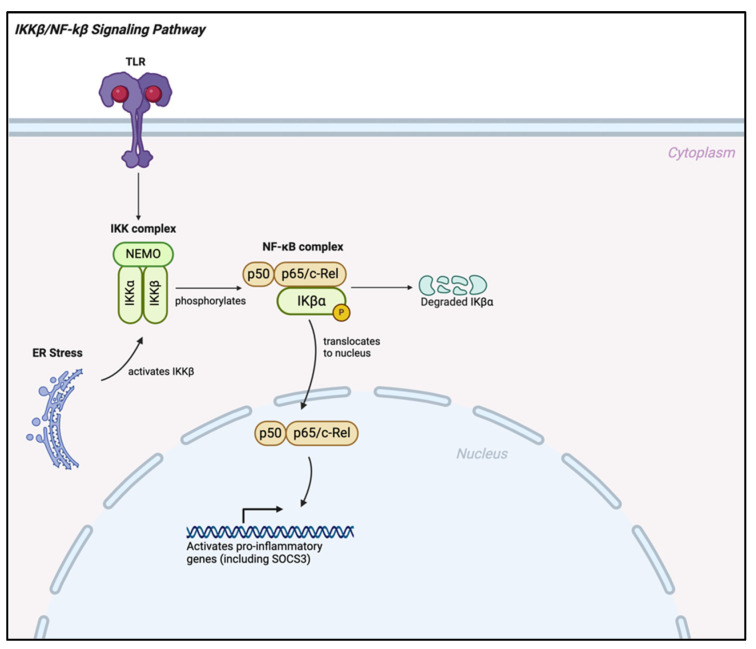
IKKβ/NF-κβ signaling pathway; TLR = Toll-like receptor; IKK = IκB kinase; NEMO = NF-κB essential modulator; IκBα = inhibitor of κB alpha; ER = endoplasmic reticulum.

**Figure 4:**
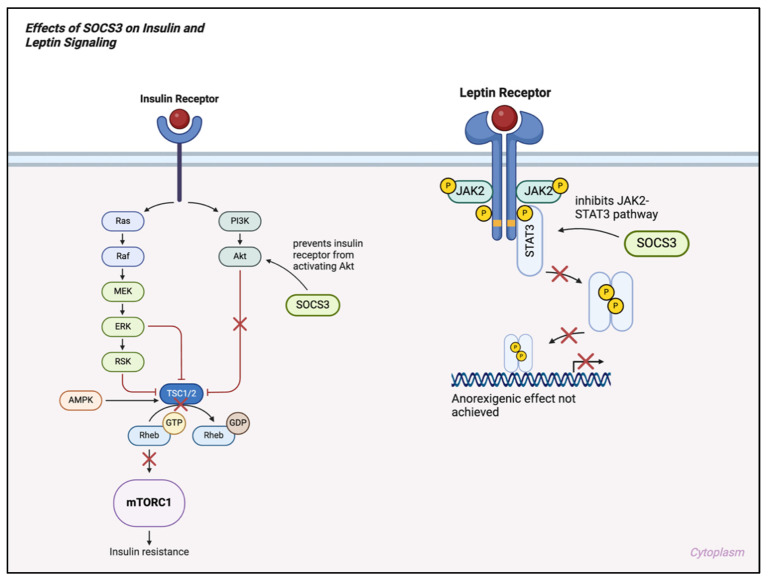
Effects of SOCS3 on insulin and leptin signaling. SOCS3 = suppressor of cytokine signaling 3; PI3K = phosphoinositide 3-kinase; Akt = protein kinase B; JAK2 = Janus kinase 2; STAT3 = signal transducer and activator of transcription 3; Ras = rat sarcoma; Raf = rapidly accelerated fibrosarcoma; MEK = mitogen-activated protein kinase; ERK = extracellular signal-regulated kinase; RSK = ribosomal S6 kinase; AMPK = AMP-activated protein kinase; TSC1/2 = tuberous sclerosis complex 1/2; Rheb = Ras homolog enriched in brain; mTORC1 = mechanistic target of rapamycin complex 1; GDP = guanosine diphosphate; GTP = guanosine triphosphate.

In neurons of the ARC, STAT3 activation enhances *POMC* gene expression and suppresses the production of AgRP/NPY [95]. STAT3 also induces SOCS3 expression, which, aside from its involvement in the IKKβ/NF-κβ signaling pathway, negatively regulates leptin signaling, generating a built-in feedback loop for controlling this pathway (Figure 3) [95,96]. In mouse models where STAT3 is selectively deleted in the hypothalamus, obesity occurs, mirroring the severe weight gain seen in leptin-deficient animals (Figure 4) [97]. Another key component in leptin signaling is the role of PTPs, which control the activity of the JAK/STAT pathway by removing phosphate groups from key tyrosine residues [95].

Like STAT3, STAT5 regulates appetite. Mice lacking STAT5 in the hypothalamus develop obesity due to increased food consumption, though the exact genetic targets responsible for this are not entirely understood. Insight into granulocyte–macrophage colony-stimulating factors, which activate STAT5, reducing appetite, corroborate its role in metabolic regulation [98].

Factors contributing to IR include impaired leptin transport to the brain, stress within neurons in the hypothalamus, elevated PTP activity, and chronic inflammation, which reduce leptin signaling in neurons of the hypothalamus [95,96,99]. Obese individuals, who normally exhibit high blood levels of leptin, bypass its effects using one or a combination of the aforementioned factors [99,100].

Paradoxically, elevated leptin levels may contribute to dysfunction in pancreatic β-cells and hypertension, though the role of leptin in these conditions remains controversial [101,102,103].

### 5.5. PPAR-γ Signaling Pathway

PPAR-γ is a ligand-activated transcription factor. Evidence suggests that agonism of PPAR-γ delays the progression and onset of neurodegenerative diseases [104]. This neuroprotective effect is achieved via the promotion of neuronal differentiation, oligodendrocyte synthesis, and axonal growth [105,106]. PPAR-γ is expressed in brain tissue and is particularly elevated in astrocytes, oligodendrocytes, neurons, and microglia [107]. PPAR-γ is also expressed in adipose tissue and, to a lesser extent, in skeletal muscle, the liver, and the heart [105]. In adipose tissue, PPAR-γ plays an essential role in regulating the metabolism of glucose and lipids, as well as adipocyte differentiation, critically regulating metabolic processes [108]. Additionally, PPAR-γ promotes insulin sensitivity, making it the primary target of thiazolidinediones in the treatment of T2DM [105,109].

Microglial polarization plays a critical role in the CNS response to metabolic stress, namely during the development of T2DM. Microglia can shift between pro-inflammatory M1 and anti-inflammatory M2 phenotypes based on the surrounding microenvironment, with the M2 phenotype particularly beneficial in mitigating T2DM-related inflammation and cognitive impairment [110,111]. PPAR-γ promotes M2 polarization by directly inhibiting NF-κB-mediated pro-inflammatory signaling and enhancing the expression of anti-inflammatory mediators such as interleukin-10 (IL-10) and transforming growth factor-beta (TGF-β) [112]. Additionally, it suppresses the differentiation of clusters of differentiation 4-positive (CD4+) T cells into T-helper (Th) 1 and Th17 subsets while promoting the development of forkhead box protein p3-positive (Foxp3+) regulatory T cells [113,114].

The release of anti-inflammatory cytokines, such as IL-4 and IL-10, creates a feedback loop that further drives microglia toward the M2 phenotype, dampening excessive inflammatory responses [115,116]. For instance, IL-4 acts through STAT6 to sustain M2 polarization, enhancing the secretion of growth factors that support neuronal repair and counteract neurodegeneration [115,117]. Moreover, IL-10 amplifies the expression of PPAR-γ and SOCS3, attenuating inflammatory responses and reinforcing the anti-inflammatory phenotype [6,118].

PPAR-γ inhibits NF-κB activation through several mechanisms. One key mechanism is its ability to interact with the IKK complex, which phosphorylates and degrades IκB proteins, as shown in [119]. By suppressing IKK activity, PPAR-γ prevents the nuclear translocation of NF-κB dimers to the nucleus, thereby reducing the expression of pro-inflammatory genes [119,120]. Also, PPAR-γ directly interacts with NF-κB and modulates the activation of nuclear factor E2-related factor 2 (NRF2), which helps reduce inflammation and oxidative stress during microglial activation [107].

Furthermore, PPAR-γ suppresses mTOR activity through its interaction with AMP-activated protein kinase (AMPK) and silent information regulator type 1 (SIRT1), both of which promote autophagy [121,122]. AMPK is activated under low-energy conditions and inhibits mTOR by phosphorylating tuberous sclerosis complex 2 (TSC2), which acts as a negative regulator of mTOR (122). PPAR-γ’s ability to interact with AMPK amplifies this inhibition, promoting energy balance and cellular homeostasis [121].

SIRT1, on the other hand, deacetylates and activates AMPK, which then inhibits mTOR signaling [123]. Additionally, SIRT1 can directly interact with PPAR-γ to modulate its activity [124]. This interaction not only regulates lipid metabolism but also influences adipocyte differentiation and fat storage [125,126]. Studies show that when SIRT1 is activated, it enhances AMPK’s inhibitory effect on mTOR, providing a coordinated response that helps manage fat accumulation and energy storage [122]. By reducing mTOR activity through PPAR-γ activation and AMPK and SIRT1 interactions, these pathways may suppress microglial activation, thus promoting an anti-inflammatory state and making PPAR-γ a vital target for therapeutic strategies.

## 6. Non-Coding RNAs

Non-coding RNAs (ncRNAs) are a group of transcripts that are not translated into proteins but rather act on target genes to modulate gene expression [127]. Here, we will explore microRNAs (miRNAs) and long non-coding RNAs (lncRNAs) and their roles in activating microglia to drive a self-sustaining neuroinflammatory cycle [128].

### 6.1. Long Non-Coding RNAs

lncRNAs are long ncRNAs of >200 nucleotides in length that serve a diverse array of functions throughout the body [129]. lncRNA Gm4419 has been identified as a key regulator of inflammation, primarily due to its strong transcriptional and post-transcriptional activation of the NF-kB signaling pathway [130]. Gm4419 has been found to be upregulated during cerebral microglial cell injury caused by oxygen and glucose deprivation/reoxygenation (OGD/R).

Previously, Gm4419 has been investigated in diabetic nephropathy, where its overexpression in mesangial cells significantly increased cell proliferation, inflammation, and fibrosis.

Gm4419 promotes NF-kB signaling by interacting with the IkBa protein and inhibiting its phosphorylation [131]. This interaction leads to the activation of NF-kB, leading to the production and release of pro-inflammatory cytokines such as tumor necrosis factor-α (TNF-α), IL-1β, and IL-6, ultimately contributing to cell apoptosis during OGD/R injury in vitro [132].

lncRNAs such as KCNQ1 opposite strand/antisense transcript 1 (KCNQ1OT1) and Lethe have emerged as key regulators in the pathogenesis of T2DM and its associated complications [133]. KCNQ1OT1, located on chromosome 11p15.5, significantly influences the progression of diabetic nephropathy by enhancing cell proliferation in high-glucose conditions [128]. It operates primarily by modulating the NF-kB signaling cascade, in turn inducing neuroinflammation [133].

Conversely, Lethe, a pseudogene of Rps15a, acts as a negative regulator of the NF-kB pathway, preventing excessive inflammation critical in diabetes-related inflammatory diseases [128]. Studies have shown that Lethe mitigates hyperglycemia-induced reactive oxygen species (ROS) generation in macrophages by targeting another miRNA, miR-34a. miR-34a upregulates Sirtuin 1 (SIRT1) expression, exhibiting an anti-inflammatory effect by deacetylating various transcription factors, including NF-kB and STAT3, and neutralizing ROS [134].

Additionally, Lethe overexpression in high-glucose-treated cells reduced NF-kB nuclear translocation, reducing ROS production and NADPH oxidase 2 (NOX2) expression. These results are consistent with the downregulation of the *Lethe* gene and upregulation of the *NOX2* gene in a mouse model of diabetic wound healing [135].

While current research has highlighted the essential roles of lncRNAs in T2DM, further investigations are needed to specifically explore their functions in hypothalamic microglial inflammation. Understanding how lncRNAs interact with microglial cells in the hypothalamus could provide insights into the neuroinflammatory processes associated with metabolic disorders. Furthermore, it is crucial to recognize that lncRNAs often interact with other miRNAs, contributing to the complex regulatory networks that govern inflammation and apoptosis.

### 6.2. MicroRNAs

miRNAs are regions of small non-coding RNAs that have an average length of approximately 22 nucleotides [136,137]. These are transcribed into primary miRNAs, later processed into precursor miRNAs, and eventually functional mature miRNAs that can interact with messenger RNA (mRNA) and silence expression [138,139].

Since individual miRNAs are regulated differently in different tissues around the body, it remains unclear whether central IR alters particular miRNAs in the hypothalamus [140,141]. Chalmers and colleagues suggest that prolonged insulin treatment in NPY neuronal models triggers the release of specific miRNAs. This response is maintained and preserved in the hypothalamus of hyperinsulinemic animals [142]. MKR mice, a model for elevated insulin levels, provide results while controlling confounding factors associated with an HFD [143]. Though further investigation is warranted, this could have implications for distinguishing between obesity- and HFD-related T2DM, as well as delineating the hyperinsulinemic development of disease in both lean and overweight individuals.

#### 6.2.1. miR-1983

Chalmers and colleagues identified that miR-1983, targeting IRβ, levels rise when insulin levels rise, contributing to IR in NPY neurons [142]. The variation in the extent of downregulation caused by insulin versus miR-1983 suggests that other mechanisms, like the lysosomal degradation of InsR, could also be involved [144]. Regardless, after 48 h of insulin treatment, miR-1983 levels were no longer elevated, despite the continued downregulation of IRβ, implying that miR-1983 might only be relevant in early stages. This phenomenon could however be explained by insulin degradation over time [142].

The MKR mouse model, which experiences constant high insulin exposure, supports the link between increased hypothalamic miR-1983 and hyperinsulinemia. While the exact mechanism behind insulin’s effect on miR-1983 is unknown, it may involve the conversion of miR-1983 from pre-Ile transformer-RNA (tRNA), which escapes repression by the RNA-binding protein La. Prolonged insulin signaling could inhibit La through Akt phosphorylation, increasing miR-1983 formation [142,145].

#### 6.2.2. miR-26

Studies using in vivo models of obesity have shown a correlation between decreased miR-26 expression and impaired insulin and glucose levels [146]. Research involving murine models of T2DM further suggests that downregulated levels of miR-26 expression are associated with reduced insulin sensitivity in pancreatic beta cells. Moreover, elevated miR-26a levels have been demonstrated to reduce glucose-stimulated insulin secretion (GSIS), which helps to mitigate hyperinsulinemia—a key factor in both T2DM and obesity. GSIS occurs in stages, requiring actin remodeling and focal adhesion, processes regulated by focal adhesion kinase (FAK), and extracellular signal-regulated kinase (ERK). Elevated miR-26a has been shown to decrease the phosphorylation of these kinases, dampening their downstream effects and thus reducing GSIS [147].

Furthermore, miR-26a is downregulated in microglia following TLR4 activation, while its presence suppresses inflammatory cytokine production [148]. Kumar et al. discovered that miR-26a regulates IL-6 production in microglial cells by directly targeting the pro-inflammatory transcription factor, ATF2. This indicates that miR026a/ATF2 signaling may regulate cytokine release in microglia. However, the overexpression of miR-26a did not impact JNK signaling, which is involved in LPS-induced TNF-a and IL-6 production. This suggests that miR-26a’s effect on reducing such inflammatory markers in activated microglia may occur through different mechanisms [149].

#### 6.2.3. miR-21

In vivo studies on miR-21 knockdown mice demonstrated reduced obesity and increased weight loss, suggesting that elevated miR-21 expression may be linked to weight gain and obesity. In these miR-21-deficient mice, levels of phosphatase and tensin homolog (PTEN) were restored [150]. While PTEN is primarily known as a tumor suppressor [151], it has also been associated with IR. One study found that individuals carrying mutated PTEN were more overweight and exhibited greater IR compared to controls. This is likely due to PTEN’s role in inhibiting the PI3K-AKT pathway, which is crucial for insulin signaling [152]. Additionally, this pathway is essential for the normal function of pancreatic β-cells in producing insulin [153]. These findings suggest a connection between miR-21, PTEN regulation, and metabolic processes related to obesity and IR.

TLR4 signaling is negatively regulated by miR-21, which carries out its effect by inhibiting NF-kB activity and enhancing IL-10 production in response to LPS [154]. Programmed cell death protein (PDCD4), a key molecule involved in microglial inflammation, is responsible for this regulatory role [112].

## 7. Therapeutics Targeting Microglia

### 7.1. Minocycline

How hypothalamic microglia can be used to promote insulin sensitivity and improve glycemia control in patients with T2DM is a topic of great interest. Minocycline (7-dimethylamino-6-dimethyl-6-deoxytetracycline), a second-generation antibiotic derived from the tetracycline class of drugs [155], has been recognized for its immune modulatory, anti-apoptotic [156], and, perhaps most importantly, its combined anti-inflammatory and neuroprotective effects [157,158,159].

Minocycline inhibits microglial activation, which is a driver of neuroinflammation [160,161]. Minocycline acts by modulating the TLR-4/NF-κB signaling pathway [162], which, through a cascade of downstream pathways, significantly reduces the release of systemic pro-inflammatory cytokines, including TNF-α, COX-2, IL-1β, and IL-6 [163,164,165]. Minocycline simultaneously activates neuroprotective pathways, particularly in relation to the nucleus tractus solitarius (NTS), where c-Fos-positive neurons play a neuroprotective role [166]. However, not much is known about its role in the hypothalamus, a primary area of interest for T2DM and associated risk factors.

A study by Valdearcos and colleagues demonstrated that the use of PLX5622, a colony-stimulating factor 1 receptor (CSF1R) inhibitor, leads to weight loss in HFD-fed mice and increases microgliosis by depleting microglia. Furthermore, HFD-fed mice who have a deletion of *Ikbkb* (IKKβ^MGKO^), encoding IKKβ, also experienced weight loss [58]. This is supported by a study by Zhang and colleagues, who reported an atypical activation of hypothalamic IKKβ/NF-κB through endoplasmic reticulum stress during overnutrition [56]. Taken together, these studies reinforce the role of microglial responses in improving the status of mice with diet-induced obesity. The study by Valdearcos and colleagues also indicates that A20^MGKO^ mice exhibit enhanced microglial activation and subsequent inflammation, contributing to the progression of obesogenic processes and metabolic dysfunctions [58].

More recently, Coker and colleagues examined the role of the pharmacological inhibition of microglial activation using minocycline on the mitigation of neuroinflammation, and they also examined the impact on glucose and insulin homeostasis. They demonstrated that minocycline not only induces weight loss in HFD-fed mice but also improves insulin sensitivity, as measured by insulin tolerance tests, and generally reduces microglial activation in the paraventricular nucleus (PVN), a region of the hypothalamus [167].

However, skepticism remains regarding the potential use of minocycline to reduce hypothalamic inflammation in T2DM. Randomized controlled trials (RCTs) assessing similar parameters to the aforementioned studies are needed to translate findings from animal to human models. Mechanistic studies are also needed to further clarify the mechanisms by which minocycline improves insulin and glucose homeostasis and attenuates microglial activation.

### 7.2. Cannabidiol

Mounting evidence from both preclinical and clinical studies has drawn attention to cannabidiol (CBD), a phytocannabinoid derived from *Cannabis sativa* L. [168], as a potential therapeutic agent in neurological disorders where neuroinflammation is implicated in the pathophysiology.

Several in vitro studies demonstrated that CBD prevents LPS-induced microglial inflammation primarily by potently inhibiting the release of pro-inflammatory cytokines, reducing ROS, and suppressing the production and release of immune cells [169,170,171]. Studies focused upon the pathways through which CBD acts to attenuate neuroinflammation collectively point toward the nuclear factor kappa B (NF-kB) signaling pathway.

In the setting of neuroinflammation, the production of ROS by NADPH oxidase is stimulated by the activation of TLR4, which leads to the activation of both the NF-kB and STAT1 signaling pathways [172]. This then leads to the release of pro-inflammatory cytokines, including interleukins, interferons, and transforming growth factors. CBD acts upon NADPH oxidase to modulate the pathway and affect its anti-neuroinflammatory function [171,173].

As these same pathways are involved in neuroinflammation related to T2DM, CBD is a potential candidate for neuro-immune modulation. However, many of these studies have been conducted on single cell lines and cannot account for crosstalk within the poorly understood neuro-immune framework. Also, these studies focus on neuroinflammation-driven neurological disorders, such as epilepsy, multiple sclerosis, and neuropathic pain [174] rather than on T2DM. Additionally, the translation of these early preclinical findings to clinical practice is still far from reality.

### 7.3. Non-Pharmacological Interventions

Non-pharmacological interventions can also mitigate the metabolic effects of microglial-driven neuroinflammation. Amongst such interventions are omega-3 fatty acids, cinnamic acid, ketogenic diets, and exercise.

#### 7.3.1. Omega-3 Fatty Acids

As noted earlier, long-chain SFAs can precipitate neuroinflammation. These effects can be reversed using other fatty acids, mainly polyunsaturated fatty acids (PUFAs). Recent evidence has shown that an HFD supplemented with fish oils reduces hypothalamic inflammation in rats while attenuating the inflammatory effects of SFAs [175,176,177]. G-protein-coupled receptors (GPCRs) GPR40/FFA1 and GPR120/FFA4 have been implicated as targets of anti-inflammatory fatty acids [178,179].

Some of the most often studied anti-inflammatory fatty acids include omega-3 fatty acids, docosahexaenoic acid (DHA), and eicosapentaenoic acid (EPA) [175,179,180,181,182,183,184]. Historically, omega-3 fatty acids reduce inflammation by reducing the production of prostaglandins PGE2 and PGF2α by interfering with the activity of microsomal prostaglandin E synthase (mPGES) and COX enzymes. Notably, omega-3 fatty acids upregulate the expression of PPAR-γ [185], though the exact mechanism by which they do so is unclear [186]. This upregulation is significant, considering that PPAR-γ regulates genes encoding glucose-6-phosphatase (G6P) and fatty acid transporter-1 (FAT-1), which are critical in metabolic processes. As such, omega-3 fatty acids have been historically associated with enhanced insulin sensitivity [187,188,189]. It is therefore plausible that omega-3 fatty acids could inhibit NF-κB pro-inflammatory signaling and promote M2 polarization via their interactions with PPAR-γ in microglia. However, further research in this area is warranted to elucidate the nature of this relationship.

A study by Salsinha and colleagues reinforced the essential role that the IKKβ/NF-κβ signaling pathway plays in metabolic-related hypothalamic inflammation. Using antagonists and agonists of GPR120/FFA4, their outcomes were two-fold. First, they demonstrated that omega-3 fatty acids diminish the activation of NF-κβ, initially induced by palmitic acid and fructose, as indicated through an increase in degraded IkBα and accumulation of the p65-NF-κβ subunit in the nucleus following translocation. This effect also extends to conjugated linoleic acid (CLA) and conjugated linolenic acid isomer (CLNA), which exhibit similar characteristics to omega-3 fatty acids. Second, they demonstrated that this anti-inflammatory effect is mediated through GPR120 within microglia [190].

These insights are of great clinical significance, suggesting that modulating GPR120 receptors within microglia could reduce hypothalamic inflammation, re-regulate metabolic processes, and improve glycemic control.

#### 7.3.2. Cinnamic Acid

One longitudinal study examined the effect of cinnamic acid (CA) on inflammatory responses and obesity-induced metabolic changes in HFD-induced obese C57BL/6 mice, clearly demonstrating its role in attenuating microglial-driven neuroinflammation. Cytokine modulation was observed with reductions in levels of TNF-α, which interferes with glucose and insulin homeostasis by blocking the downstream IRS-1/AKT kinase-mediated insulin signaling pathway. The HFD feeding model also demonstrated a decrease in macrophage accumulation within the hypothalamus, which would normally contribute to low-grade inflammation characteristic of early stages of metabolic disease [160,191].

CA generally decreased appetite, weight gain, and fat accumulation, owing to a reduction in hypothalamic inflammation [192]. For such reasons, it may be useful in therapeutics or as a supplement to treat metabolic disorders.

#### 7.3.3. Ketogenic Diets

Ketogenic diets (KDs) consist of high amounts of fat, adequate protein, and little-to-no carbohydrates [193,194]. Historically, they have been employed since the early 1920s to treat epilepsy patients, remarkably reducing seizure frequency for reasons that were unclear at the time [193,195,196,197].

KDs have recently been linked to improvements in brain metabolism and the remission of neurodegenerative conditions [198,199], as well as a reduction in psychological stress responses [200]. Most studies that examined the neuroprotective role of ketogenic interventions involved patients with either Alzheimer’s disease (AD) or Parkinson’s disease (PD) [198,199].

This neuroprotective role has been linked to the production of β-hydroxybutyrate (BHB), a ketone body produced in large quantities by hepatic mitochondria when a KD is consumed [201]. BHBs can easily cross the BBB either via transporters or by simple diffusion, enabling their neuroprotective role in the brain [202,203]. The mechanisms by which BHBs carry out this role, however, are not clear.

Zhang and colleagues suggest that BHBs may indirectly interact with PPAR-γ to ameliorate IR. The group identified that BHBs act through a hydroxycarboxylic acid receptor 2 (HCAR2)/Ca^2+^/cyclic adenosine monophosphate (cAMP)/protein kinase A (PKA)/rapidly accelerated fibrosarcoma-1 proto-oncogene serine/threonine-protein kinase (Raf1)/extracellular signal-regulated kinases 1/2 (ERK1/2) pathway, ultimately inhibiting PPAR-γ, to achieve this effect [204]. It is, however, unclear whether these findings can be translated to microglial PPAR-γ.

BHBs have also been shown to inhibit NLRP3 inflammasomes [205], decrease cytokine release [206], and decrease the production of ROS by increasing the oxidation of NADH [206,207,208], among other mechanisms.

They also influence microglial activity, with a 2014 study showing that BHBs significantly reduced the release of pro-inflammatory cytokines iNOS, COX-2, TNF-*α*, IL-1*β*, and IL-6 in LPS-induced BV2 cells. Unsurprisingly, this effect was mediated via inhibition of the IKKβ/NF-κβ signaling pathway, which is involved in metabolic changes related to T2DM [203].

Another study [209] sought to evaluate the overall effects of BHBs on LPS-induced BV2 cells rather than only measuring levels of pro-inflammatory proteins. This study found that BHBs can modulate the polarization of BV2 cells to a pro-inflammatory M2 phenotype [210], hence reducing the cell’s migratory capacity and the production of cytokines, typically characteristic of microglial activation [27,53,198].

The findings are promising, given that BHB affects pathways implicated in metabolic disorders, including T2DM. Classically, KDs improve T2DM by reducing the glycemic response to carbohydrates [211,212], but perhaps the role of KDs should be explored further in improving glycemic control from an anti-neuroinflammatory approach.

#### 7.3.4. Other Non-Pharmacological Interventions

There are other non-pharmacological interventions that mitigate microglial-driven neuroinflammation that are worth highlighting. The immunomodulatory effects of these interventions have been demonstrated in neurodegenerative diseases such as AD, PD, multiple system atrophy (MSA), frontotemporal dementia (FTD), amyotrophic lateral sclerosis (ALS), Huntington’s disease (HD), and progressive supranuclear palsy (PSP) [25].

Many of these are nutritional in nature and include carotenoids, such as lycopene, which demonstrated antineuroinflammatory effects in vitro by inhibiting COX-2 expression after BV-2 microglial cells were treated with LPS [213]. Interestingly, typical CNS inflammation in T2DM, leading to diabetic retinopathy, can be mitigated using carotenoids. Astaxanthin and lutein, for instance, reduce the activity of the NF-κB signaling pathway, hence reducing the release of pro-inflammatory markers [214,215,216], as detailed in Section 5.1.

Sulfur compounds, such as sulforaphane, decrease the expression of IL-1β, IL-6, and TNF-α in vitro [217]. These have been suggested as adjuvants to the treatment of T2DM in the past, given that they attenuate oxidative stress and reduce the formation and accumulation of reactive intermediates [218]. Perhaps this effect extends to microglia, where the aforementioned cytokines are released in a setting of oxidative stress. Other bioactive compounds suggested to have immunomodulatory effects, though with weaker circumstantial evidence, include phytosterols [219,220,221], where investigations are minimal, and phenolic compounds [222,223,224], where evidence is contradictory [225].

Furthermore, the microglial phenotype can be targeted using vitamin D, which attenuated neuroinflammation in an animal model of PD by promoting M2 polarization [226]. Previous experimental and epidemiological evidence suggests that vitamin D deficiency is associated with T2DM via a reduction in insulin secretion by β-cells [227]. Bridging these findings suggests that vitamin D may improve insulin release and maintain β-cell mass by protecting microglia from inflammation and oxidative stress.

However, it is important to remember that the points highlighted in this section are largely speculative and require experimental evidence to be substantiated. Additionally, the therapeutic strategies discussed here are not validated in humans and rely on animal studies to demonstrate their antineuroinflammatory effects. It would, however, be interesting to see how these strategies translate to the care of T2DM patients.

## 8. Future Perspectives

Emerging knowledge regarding the role of hypothalamic microglia in the pathogenesis of T2DM is intriguing, for which reason we compiled the studies we referred to in Supplementary Tables S1–S3, referring to in vitro studies, in vivo studies, and clinical trials, respectively. However, much remains to be explored. Future research should focus on exploring the mechanistic pathways by which microglial activation accelerates the onset of systemic IR and eventually T2DM. Existing evidence implicates the IKKβ/NF-κβ signaling pathway as central to energy imbalance, and metabolic disturbance though a direct relationship between this pathway and IR has not yet been established. Therefore, it might be of interest to explore this relationship in HFD rat models after IR is achieved or, alternatively, using genetically or spontaneously induced T2DM rat models.

Furthermore, microRNAs that help regulate hypothalamic inflammation can be investigated not only as therapeutic targets but also as diagnostic and prognostic ones. Previously, dysregulated serum miRNAs implicated in glucose homeostasis were studied as diagnostic biomarkers in prediabetic patients and yielded comparable or possibly better predictive values than traditional indices [228,229]. Perhaps these should be explored further with regard to hypothalamic inflammation.

A pressing question is how to target hypothalamic microglia to alleviate IR. A number of different therapeutics that inhibit microglial activation, including minocycline and cannabidiol, have been identified amongst other non-pharmacological interventions. These interventions may prove useful as part of the shift toward personalized medicine, where the interplay between genetic, epigenetic, and environmental factors are integral to the design of patient care, especially for chronic multifactorial diseases such as T2DM.

## 9. Conclusions

There is an established association between microglial activation and neurodegenerative and neuroinflammatory diseases like AD and PD. More recent evidence points to hypothalamic inflammation, mediated by microglia, as being a driver of metabolic dysregulation, particularly IR and T2DM. The identification of signaling pathways that underlie this association paves the way for the consideration of therapeutic options to alleviate microglial activation and its negative downstream effects on metabolic homeostasis, offering a potentially novel approach to address the burgeoning epidemic of T2DM.

## Data Availability

Not applicable.

## Supplementary Materials

The following supporting information can be downloaded at: https://www.mdpi.com/article/10.3390/ijms252313169/s1.
