# Supplementary material for: The Role of Hypothalamic Microglia in the Onset of Insulin Resistance and Type 2 Diabetes: A Neuro-Immune Perspective
Source: Int J Mol Sci. 2024 Dec 7;25(23):13169. doi: 10.3390/ijms252313169 (PMC11642714; doi:10.3390/ijms252313169)
Supplement: Supplementary file 1 [file ijms-25-13169-s001.zip › ijms-3335926-supplementary.pdf]

Supplementary Materials

S1: In Vitro Studies

Author	Year	Country	Samples	Intervention	Main Findings
Yang et al. (51)	2022	Dalian, China	Male C57BL/6J mice	HFD	Long-term HFD provokes insulin resistance in mice and causes central and peripheral nervous system hyperinsulinemia.
Zhuang et al. (7)	2017	Zhejing, China	C57BL/6 J mice	Arachidonic acid (AA)	AA affects obesity likely through a gut-hypothalamus-adipose-liver axis.
De Solis et al. (10)	2024	Cologne. Germany	C57BL/6 J mice	HFD	HFD-induced obesity may impair the ability of AgRP and POMC neurocircuits to cooperatively increase and prolong food intake.
Ishijima et al. (26)	2021	Tokyo, Japan	Male Wistar rats	Lipopolysaccharide (LPS)	Microglia in vitro were verified to have potencies sufficient to induce all three kinds of inflammatory cytokines (TNFa, IL-1b, IL-6) in response to LPS.
Haas et al. (52)	2020	Groningen, The Netherlands	C57Bl/6 mice pups	Insulin infusions	Microglia in young mice can respond to insulin, but responsiveness is lost with aging.
Kawanokuchi et al. (53)	2007	Nagoya, Japan	C57BL/6J mice cell cultures	LPS, TGF-b, IL-17 and IL-23	Treatment with IL-17 upregulated microglial production of IL-6, macrophage inflammatory protein-2, nitric oxide, adhesion molecules, and neurotrophic factors.
Spielman et al. (54)	2015	British Colombia, Canada	Human monocytic THP-1 cell line	Insulin infusions	Insulin promotes the secretion of certain cytokines (such as IL-6 from human astrocytes and IL-8 from both human astrocytes and microglia), while suppressing the release of others (like MCP-1 from human microglia and cytotoxins from monocytic cells).
Valdearcos et al. (58)	2017	California, USA	PLX5622-treated mice	HFD	Depleting microglia significantly reduced weight gain in mice fed a HFD but not in mice fed a standard low-fat chow diet.
Ueki et al. (63)	2004	Massachusetts, USA	Male C57BL/6 mice	LPS	SOCS-1 and SOCS-3 are increased in insulin-resistant states, such as endotoxemia and obesity.
Zabolotny et al. (80)	2008	Massachusetts, USA	*Ob*/*ob* mice tissues and Zucker rats	Diet induced obesity (HFD)	Increased TNFα expression in adipose tissue accompanies PTP1B overexpression in insulin- and leptin-target tissues of mice with DIO. Activation of NFκB p65 by TNFα in 3T3-L1 adipocytes in vitro and in mouse liver in vivo leads to the recruitment of p65 to the PTP1B promoter.
Wellhauser et al. (182)	2014	Ontario, Canada	Hypothalamic cell line from the embryonic rat, rHypoE-7	Treatment of rHypoE-7 with docosahexaenoic acid (DHA), an omega-3 fatty acid.	When exposed to TNFα, the rHypoE-7 cells show an inflammatory response at the gene and the protein levels. They express high levels of GPR120, which is responsive to DHA. Pretreatment with DHA prevents the inflammatory response. This effect is lost when GPR120 levels are reduced.
Chalmers et al. (142)	2022	Toronto, Canada	mHypoE-46 and mHypoA-NPY/GFP cell line	Metformin	miR-1983 is upregulated in insulin-resistant hypothalamic neurons and targets the insulin receptor β subunit, reducing its protein levels. Metformin treatment normalized miR-1983 levels in cell cultures.
Sheedy et al. (154)	2010	Dublin, Ireland	Cell cultures (RAW 264.7 cells)	LPS	miR-21 targets PDCD4, a proinflammatory tumor suppressor, and thereby adjusts TLR4 signaling. This lowers the production of proinflammatory cytokines and increases IL-10 levels while decreasing NF-κB activity.
Giuliani et al. (157)	2005	Alberta, Canada	Blood of healthy volunteers	Minocycline	Minocycline disrupts T cell interactions with U937 cells and microglia, leading to higher TNF-α levels. It also increases IL-10 levels. It also lowers CD40L expression on T cells, impairing their ability to activate microglia, which ultimately decreases TNF-α regulation.
Henry et al. (163)	2008	Ohio, USA	BV-2 microglia cell cultures	Minocycline	Minocycline reduces LPS-triggered cytokine production in BV-2 microglia and TLR2 expression, aids in recovery from LPS-induced illness, reduces neuroinflammation, and lowers IL-6 levels in plasma
Pang et al. (165)	2012	Maryland, USA	Human circulating monocytes	LPS, minocycline	Minocycline reduced inflammation in LPS-challenged monocytes by lowering TNF-α, IL-1β, IL-6, and COX-2 levels. It also inhibited LOX-1, NF-κB, and the p38 MAPK and PI3K/Akt pathways linked to LPS activation.
Han et al. (170)	2009	Seoul, South Korea	Human monocytes	Selective agonists	CB1 receptor activation enhances pro-inflammatory responses in macrophages by increasing ROS production, while CB2 receptor activation, via Rap1, counteracts this effect. This suggests that inhibiting CB1 while selectively activating CB2 may help reduce inflammation.

S2: In Vivo Studies

Author	Year	Country	Samples	Intervention	Main Findings
Valdearcos et al. (5)	2014	California, USA	C57BL/6 mice	Enteric saturated fatty acid (SFA)	Excessive SFA intake activates microglia in the hypothalamus, independent of caloric intake, leading to neuronal stress. Palmitic acid treatment induces microglial-driven inflammation in hypothalamic cultures.
Baufeld et al. (6)	2016	Berlin, Germany	Male C57Bl/6 J mice aged 100-120 days	HFD or LFD	Long-term HFD exposure leads to hypothalamic gliosis in obese mice, without involvement of peripheral myeloid cells in the brain's immune response. After prolonged exposure, microglia in the hypothalamus shift from a pro-inflammatory to an anti-inflammatory state, no longer producing cytokines in response to plasma.
Zhuang et al. (7)	2017	Zhejiang, China	Four-week-old C57BL/6J mice	HFD enriched with AA or a continuous HFD	Arachidonic acid increases obesity, inflammation, and insulin resistance in male mice by altering gut microbiota and promoting hypothalamic inflammation, while in female mice, it improves insulin sensitivity and reduces obesity-related issues by promoting anti-inflammatory gut microbiota.
De Solis et al. (10)	2024	Germany and USA	C57BL/6 mice	Chemogenetic manipulation of AgRP and POMC neurons in mice using DREADDs	Simultaneous activation of AgRP neurons and inhibition of POMC neurons increased food intake. A specific neurocircuit involving Npy1R-expressing neurons in the paraventricular nucleus of the hypothalamus was identified as integrating signals from both AgRP and POMC neurons to regulate feeding and metabolism.
Dodd et al. (16)	2018	Melbourne, Australia	Hypothalamic POMC neurons of *Pomc*-Cre mice	Enzyme TCPTP	Blocking TCPTP improves insulin's ability to activate POMC neurons, reducing liver glucose production and improving overall glucose metabolism. In obesity, higher levels of TCPTP make insulin less effective, leading to increased liver glucose production and insulin resistance.
Nimmerjahn et al. (21)	2005	Heidelberg, Germany	C57BL/6 mice	Two-photon imaging	Microglial cells were found to be highly dynamic even in their resting state, continuously monitoring their surroundings with active processes.<br>Upon injury, such as blood-brain barrier disruption, microglia quickly shifted from a patrolling behavior to an active protective role at the site of injury
Tremblay et al. (22)	2010	Pennsylvania, USA	C57BL/6 mice	Dark adaptation and subsequent re-exposure to light	Microglial interactions with synapses in the visual cortex are dynamically modulated by sensory experience, with microglia promoting the modification or elimination of synapses during sensory deprivation, a process that is reversible with light re-exposure.
Ishijima et al. (26)	2021	Tokyo, Japan	Male Wistar rats (8 weeks old)	LPS	Inflammatory cytokines including TNFα, IL-1β, and IL-6 in response to LPS. Each inflammatory cytokine was induced by a specific combination of mitogen activated protein kinases (MAPKs).
Honka et al. (35)	2013	Turku, Finland	28 pigs (6 to 8 months old)	HFD	The HFD led to a deterioration of plasma glucose and cholesterol levels. Intestinal insulin resistance occurs in obesity before the deterioration of systemic glucose tolerance.
Shin et al. (47)	2017	New York, USA	AgRP or POMC mice	HFD	Insulin signaling in POMC neurons inhibits lipolysis and contributes to hepatic steatosis after a HFD, whereas signaling in AgRP neurons regulates hepatic glucose production without affecting lipolysis.
Thaler et al. (48)	2012	Washington, USA	C57BL/6 mice and rats	HFD	Reactive gliosis and neuronal injury markers were detected in the hypothalamic arcuate nucleus of rats and mice within a week of a high-fat diet.
Yang et al. (51)	2022	Dalian, China	C57BL/6J mice (4 weeks old)	HFD	Hyperinsulinemia may induce M1 activation and proliferation of microglia due to altered energy metabolism in insulin-resistant conditions.
Haas et al. (52)	2020	Groningen, The Netherlands	3 and 22-month-old male Wistar rats	Insulin infusions	Intracerebroventricular insulin injections increased microglial activation (CD68+ cells) and increased COX-2 and IL-1β levels in the hippocampus.
Seeger et al.(150)	2014	Frankfurt, Germany	11 db/db mice	Locked nucleic acid-modified anti-miRs directed against miR-21 (LNA-21)	Suppression of miR-21 in the heart and white adipose tissue led to reduced body weight, smaller adipocytes, and lowered serum triglycerides, without impacting cardiac function or causing toxicity. miR-21 inhibition increased expression of targets like TGFBR2 and PTEN in white adipose tissue.
Ueki et al. (63)	2004	Massachusetts, USA	8-week-old male C57BL/6 mice	LPS	In insulin-resistant states like endotoxemia and obesity, SOCS-1 and SOCS-3 are elevated, binding to different IR domains and inhibiting IRS-1 and IRS-2 phosphorylation without affecting IR tyrosine phosphorylation.
Zabolotny et al. (80)	2008	Massachusetts, USA	*Ob*/*ob* mice tissues and Zucker rats	HFD	Increased TNFα expression in adipose tissue is associated with PTP1B overexpression in insulin- and leptin-target tissues. TNFα activates NFκB p65 in 3T3-L1 adipocytes and mouse liver, promoting p65 recruitment to the PTP1B promoter.

White et al. (81)	2009	Indiana, USA	*ob*/*ob* mice on the C57BL6 background	HFD	High-fat diets induce hypothalamic leptin resistance and increase PTP1B expression, independent of chronic hyperleptinemia.
Bence et al. (82)	2006	Massachusetts, USA	Mice	PTP1B deletion	Neuronal PTP1B deficiency increased leptin sensitivity and improved glucose regulation.
Cheng et al (158)	2015	Jiangsu, China	Dicer conditional knockout (cKO) mice	Minocycline	While 2 months of minocycline reduced neuroinflammation, it did not prevent neuronal or synapse loss, nor did it inhibit apoptosis or reduce tau hyperphosphorylation.
Qaid et al. (162)	2024	Kelantan, Malaysia	Adult male Sprague Dawley (SD) rats	LPS and monocycline	Minocycline (50 mg/kg) significantly reduced phosphorylated tau protein levels and decreased TLR-4, NF-κB, TNF-α, and COX-2 positive cells in various hippocampal regions of the LPS group, showing greater effectiveness than memantine and lower doses of minocycline (25 and 10 mg/kg)
Bassett et al. (164)	2021	Tianjin, China	Male C57BL/6 mice	Minocycline	Minocycline reduced depressive-like behaviors and memory deficits caused by chronic stress. It normalized microglial activation, decreased CD68-expressing microglia, and restored neurogenesis in the hippocampus, particularly in the dentate gyrus and CA2 regions.
Xiu et al. (166)	2021	Beijing, China	Male Sprague Dawley rats	Minocycline	Minocycline significantly mitigates neuroinflammation induced by LPS through the activation of the nucleus of the solitary tract.
Coker et al. (167)	2022	Pennsylvania, USA	Male C57Bl/6J mice	HFD, and HFD + minocycline-treated groups	Minocycline lowered body mass and fat in HDF mice without affecting their food intake, improved insulin sensitivity, reduced hyperinsulinemia., prevented microglial activation, and reduced inflammation in the PVN.
de Mello et al. (177)	2019	Brazil	Male Swiss mice	Omega-3 (fish oil)	Omega-3 treatment reduced visceral fat and partially reversed inflammation, oxidative damage, and mitochondrial dysfunction in the brains of obese animals, indicating its potential as a beneficial intervention in obesity management without affecting overall body weight.
Oh et al. (179)	2010	California, USA	30 C57BL/6 mice	Omega-3 fatty acid	Omega-3 fatty acid improved insulin sensitivity, glucose infusion rates, and skeletal muscle insulin sensitivity while reducing hepatic glucose production in wild-type mice, but had no effect on knockout mice, confirming that these benefits are mediated by GPR120.
Lee et al. (191)	2022	Seoul, South Korea	Male C57BL/6 mice	Cinnamic Acid (CA)	CA reduces activated microglia and astrocytes in the hypothalamus while inhibiting TNF in the bloodstream, potentially affecting microglial activation in the thalamus. Given that hypothalamic inflammation is linked to increased appetite and weight gain, this anti-inflammatory agent may enhance insulin resistance.
Oh et al. (181)	2014	California, USA	C57BL/6 WT mice	HFD containing 30 mg/kg of the synthetic GPR120 agonist cpdA	GPR120 cpdA significantly improved glucose tolerance, insulin tolerance, and insulin sensitivity in wild-type mice without affecting body weight, while also increasing glucose infusion rates. CpdA had no effect on GPR120 knockout mice, indicating that these metabolic benefits are mediated through GPR120.
Saponaro et al. (84)	2012	Milan, Italy	Chick embryo	LPS	First evidence of the activation of pAkt by LPS occurs before NF-κB activation, suggesting that PI3K/Akt is involved in the NF-κB-dependent inflammatory responses of microglia.
Ono et al. (87)	2008	New York, USA	Male Sprague-Dawley rats	HFD	Constitutive activation of S6K in the medio basal hypothalamus mimicked the effects of HFD in rats. Inhibition of S6K restored insulin's ability to suppress hepatic glucose production after HFD, suggesting that hypothalamic S6K activation contributes to hepatic insulin resistance in response to short-term nutrient excess.
Lee et al. (98)	2008	Maryland, USA	Nestin-Cre transgenic mice	Stat5 gene deletion, by Cre-mediated recombination	Mice with CNS-specific deletion of the Stat5 gene developed significant obesity, characterized by increased body weight, elevated serum leptin levels, and reduced SOCS3 expression in the arcuate nucleus. Following a ketogenic diet, their microglia showed fewer cellular stress markers.
Vinnikov et al. (141)	2014	Heidelberg, Germany	C57BL/6N mice	Dicer1 or Pten gene disruption	Mice with Dicer1 gene deletion in the ARC of the hypothalamus developed hyperphagic obesity due to chronic overactivation of the PI3K-Akt-mTOR signaling pathway. The loss of Dicer-dependent microRNAs contributed to the obesity phenotype.
Héron-Milhavet et al. (143)	2004	Maryland, USA	MKR/CD36 mice	CD36	The MKR/CD36 double-transgenic mice exhibited normalization of hyperglycemia and hyperinsulinemia, improved liver insulin sensitivity, and restored beta-cell function, suggesting that insulin resistance in MKR mice is linked to increased muscle triglyceride levels, which can be partially reversed by reducing these levels.

Gzielo et al. (202)	2019	Krakow, Poland	Male Wistar rats	Ketogenic diet (KD)	Microglia in KD-fed animals exhibited increased branching, but no significant changes in glial activation were noted compared to ND controls, likely due to a lack of pathology. KD is associated with reduced microglial inflammation.
Julio-Amilpas et al. (206)	2015	Mexico City, Mexico	Male Wistar rats	Beta-hydroxybutyrate	Systemic administration of D-BHB significantly reduces reactive oxygen species (ROS) production and prevents neuronal death in hypoglycemic animals by stimulating ATP production and demonstrating protective effects that extend beyond its metabolic actions, making it a promising candidate for treating ischemic and traumatic injuries
Pimentel et al. (183)	2012	São Paulo, Brazil	Male Wistar rats	HFD with soy oil and fish oil	HFD with soy oil elevated leptin levels, and impaired insulin response. Those on a fish oil diet had lower fat, improved lipid profiles, and maintained insulin sensitivity. This indicates that dietary fat type influences hypothalamic insulin signaling and food intake.
Cintra et al. (184)	2012	Campinas, Brazil	Male Wistar rats and Swiss mice	Flaxseed oil and olive oil	In a mouse model of diet-induced obesity, partially substituting dietary fats with flaxseed oil or olive oil improved hypothalamic inflammation, insulin resistance, and body adiposity, while also enhancing expressions of POMC and CART, and activating the GPR120 receptor in the hypothalamus.
Talukdar et al. (180)	2011	California, USA	GPR120 knockout (KO) mice	HFD containing omega-3	Omega-3 supplementation improved glucose tolerance in wild-type mice compared to both groups on a high-fat diet, while it had no effect on GPR120 knockout mice.

S3: Clinical Trials

Author	Year	Country	Study Design	Sample Size	Phase	Intervention	Main Findings
Cukras et al. (161)	2014	USA	Open-label interventional study	6	Completed	Minocycline	Minocycline was well tolerated and not associated with significant safety issues. Minocycline has shown to decrease activation of retinal microglia, thus improving the outcomes of patients with diabetic macular edema (DME).
Honka et al. (35)	2013	Turku, Finland	Observational	240	2	Fasting state vs. euglycemic hyperinsulinemia	Obese patients had significantly lower insulin-mediated whole-body glucose uptake (GU) than controls. Healthy volunteers showed a two- to threefold increase in intestinal GU with hyperinsulinemia, while obese patients had comparable fasting intestinal GU (positive correlation).
Jimenez-Lucena et al. (229)	2018	Córdoba, Spain	Randomized Control Trial	462	2	Plasma circulating miRNAs profile	Dysregulated plasma levels of miR-150, miR-30a-5p, miR-15a, and miR-375 were observed years before the onset of T2DM and pre-DM and could be used to assess a patient's risk of developing DM. This can improve prediction of DM onset and prevention of T2DM in high-risk patients.
